# TRIF-TAK1 signaling suppresses caspase-8/3-mediated GSDMD/E activation and pyroptosis in influenza A virus-infected airway epithelial cells

**DOI:** 10.1016/j.isci.2024.111581

**Published:** 2024-12-12

**Authors:** Yuling Sun, Huidi Yu, Zhihao Zhan, Wei Liu, Penggang Liu, Jing Sun, Pinghu Zhang, Xiaoquan Wang, Xiufan Liu, Xiulong Xu

**Affiliations:** 1 College of Veterinary Medicine, Institute of Comparative Medicine, Yangzhou University, Yangzhou 225009, Jiangsu Province, P.R. China; 2 Institute of Translational Medicine, Yangzhou University Medical College, Yangzhou 225009, Jiangsu Province, P.R. China; 3 Animal Infectious Disease Laboratory, College of Veterinary Medicine, Yangzhou University, Yangzhou 225009, China; 4 Jiangsu Co-innovation Center for Prevention and Control of Important Animal Infectious Diseases and Zoonosis, Yangzhou University, Yangzhou 225009, Jiangsu Province, China

**Keywords:** molecular network, virology, cell biology, integrative aspects of cell biology, model organism

## Abstract

Pyroptosis plays an important role in attracting innate immune cells to eliminate infected niches. Our study focuses on how influenza A virus (IAV) infection triggers pyroptosis in respiratory epithelial cells. Here, we report that IAV infection induces pyroptosis in a human and murine airway epithelial cell line. Mechanistically, IAV infection activates caspase-8 and caspase-3, which cleave and activate gasdermin (GSDM) D and GSDME, respectively. Z-nucleic acid-binding protein 1 (ZBP1) and receptor-interacting protein kinase (RIPK) 1 activity but not RIPK3 are required for caspase-8/3 and GSDMD/E activation and pyroptosis. GSDMD/E, ZBP1, and RIPK1 knockout all block IAV-induced pyroptosis but enhance virus replication. Transforming growth factor β-activated kinase 1 (TAK1) activation via the adaptor protein TRIF suppresses RIPK1, caspase-8/3, and GSDMD/E activation and pyroptosis. The TAK1 inhibitor 5Z-oxzeneonal (5Z) enhances IAV-induced caspase-8/3 and GSDMD/E cleavage in the lung tissues of IAV-infected mice. Our study unveils a previously unrecognized mechanism of regulation of IAV-induced pyroptosis in respiratory epithelial cells.

## Highlights

• IAV infection activates caspase-8/3 and GSDMD/E to induce pyroptosis
• ZBP1 and RIPK1 but not RIPK3 are required for caspase-8/3 activation
• TRIF-TAK1 signaling blocks IAV-induced RIPK1 activation and pyroptosis
• The TAK1 inhibitor 5Z-oxzeneonal enhances IAV-induced pyroptosis *in vivo*

## Graphical abstract

**Figure:**
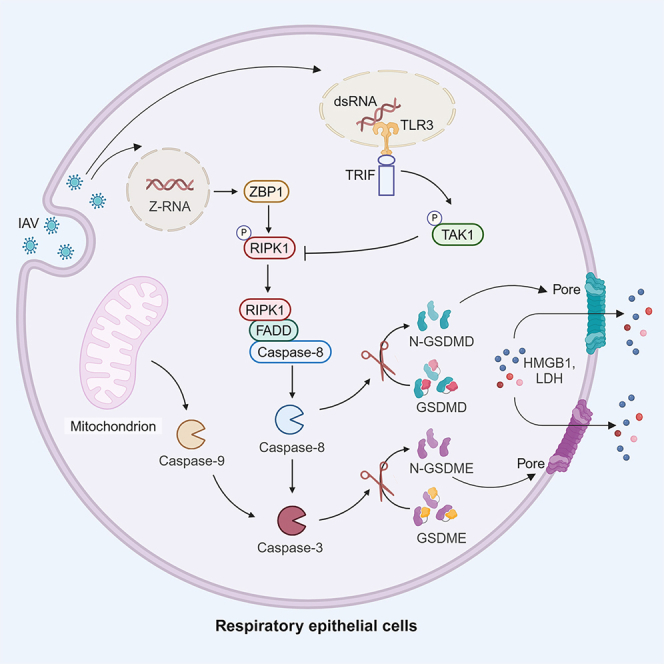

## Introduction

Influenza is a highly contagious infectious disease of the respiratory tract caused by the influenza virus. Influenza A virus (IAV) H1N1 and H3N2 subtypes have triggered four pandemics since the beginning of the 20th century.^1^ Both subtypes also cause seasonal epidemics, resulting annually in about 500,000 deaths worldwide and posing a serious threat to global public health.^1^^,^^2^ IAV is a segmented single-stranded RNA virus that primarily infects the epithelial cells of the respiratory tract. IAV can also infect some immune cell types, including monocytes, macrophages, and antigen-presenting cells, and elicit strong antiviral immune responses that restrain virus replication.^3^ IAV-infected cells commit “suicide” or programmed cell death to reduce virus replication.^4^^,^^5^^,^^6^ It is of great importance to understand the molecular mechanisms of antiviral immunity and programmed cell death, particularly in the host cells of influenza A virus, e.g., the respiratory epithelial cells.

IAV infection-induced cell death includes apoptosis, necroptosis, and pyroptosis.^4^^,^^5^^,^^6^^,^^7^^,^^8^ In murine embryonic fibroblasts (MEFs), ZBP1 (Z-nucleic acid-binding protein 1), a Z-nucleic acid binding protein, binds the viral RNA of IAV and activates the receptor-interacting protein kinase 3 (RIPK3). RIPK3 then phosphorylates and activates MLKL (mixed lineage kinase domain-like) to induce necroptosis.^9^^,^^10^^,^^11^ It is widely accepted that IAV induces necroptosis via the ZBP1-RIPK3-MLKL pathway.^4^^,^^12^ In addition, RIPK3 interacts with RIPK1 to engage FADD (FAS-associated protein with death domain) and caspase-8 to induce apoptosis.^9^^,^^10^^,^^11^ RIPK1 functions as an adaptor protein; its kinase activity is dispensable for IAV-induced apoptosis in MEFs.^9^^,^^10^^,^^11^ IAV induces MEF apoptosis via the ZBP1-RIPK3-RIPK1-FADD-caspase-8 pathway.^4^^,^^12^ Pyroptosis is another form of programmed cell death orchestrated by the gasdermin (GSDM) superfamily and plays a crucial role in inflammation.^13^ The molecular mechanisms of IAV-induced pyroptosis have been largely studied in murine bone-marrow-derived macrophages (BMDMs). IAV activates the NLRP3 (NOD-, LRR-, and pyrin domain-containing protein 3) inflammasome and caspase-1, which cleaves GSDMD and pro-interleukin (IL)-1β.^14^ The truncated N-terminal domain of GSDMD oligomerizes and forms pores in the plasma membrane, causing the release of inflammatory factors such as HMGB1 (high-mobility group box 1), LDH (lactate dehydrogenase), IL-1β, and ATP.^15^^,^^16^ ZBP1 is required for IAV-induced pyroptosis. ZBP1 initiates the assembly of a complex termed “PANoptosome,” which includes RIPK3, RIPK1, FADD, caspase-8, and caspase-6.^17^^,^^18^ This super complex then activates the NLRP3 inflammasome. Lee et al. recently reported that NLRP3 is indirectly activated by K+ efflux through MLKL pores after BMDMs undergo necroptosis.^19^ In contrast, Guy et al.^20^ reported that protein kinase R (PKR) activation by IAV leads to caspase-8/3 and GSDME activation and pyroptosis in normal human bronchial epithelial (NHBE) cells. How GSDME is activated and whether GSDMD is also involved IAV-induced pyroptosis in airway epithelial cells remain controversial. Whether IAV induces pyroptosis in epithelial cells via a mechanism different from that in macrophages remains to be investigated.

TRIF (TIR domain-containing adapter-inducing interferon-β) is an adaptor protein of the Toll-like receptor (TLR) signaling pathway. In addition to its role in activating TLR-mediated innate immunity, TRIF is involved in the assembly of a multiprotein complex termed “TRIFosome.”^21^^,^^22^^,^^23^ This complex contains TRIF, ZBP1, RIPK1, FADD, and caspase-8 and functions to mediate lipopolysaccharide (LPS)-induced apoptosis and pyroptosis.^21^^,^^22^^,^^23^ Transforming growth factor β-activated kinase 1 (TAK1) is a serine/threonine kinase downstream of TRIF and plays an important role in activating several kinases such as IKK, JNK, and p38, which mainly function to regulate the expression of inflammatory cytokines but can also counteract cell death in various settings.^24^^,^^25^^,^^26^ For example, inhibition of TAK1 activity by the YopJ protein of *Yersinia* bacteria or by a TAK1 inhibitor in LPS-treated BMDMs enhances GSDMD activation and pyroptosis.^23^^,^^27^^,^^28^ Whether TRIF and TAK1 are involved in regulating influenza virus-induced pyroptosis remains unexplored. Here, we report that IAV induces pyroptosis in airway epithelial cells by activating caspase-8 and caspase-3, which cleave and activate GSDMD and GSDME, respectively. TAK1 activation through TRIF attenuates IAV-induced pyroptosis by suppressing RIPK1 activation. Our study provides evidence that IAV induces pyroptosis via a pathway that is distinct from what is known in macrophages.

## Results

### IAV induces NLRP3-independent pyroptosis

**Figure 1:**
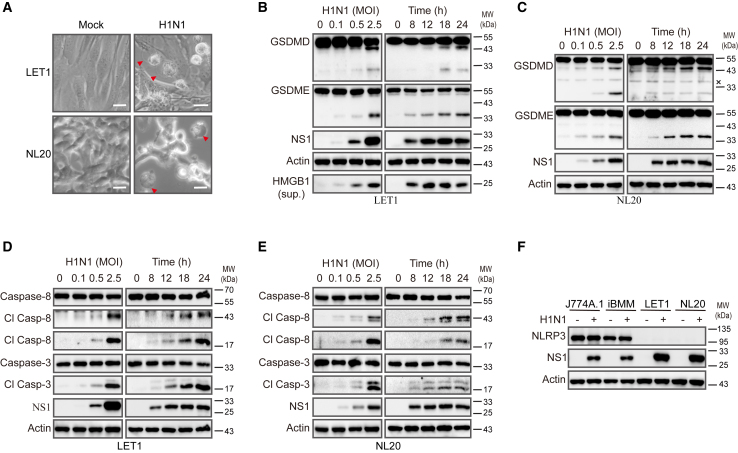
IAV induces pyroptosis in airway epithelial cells (A) LET1 and NL20 cells were infected with 2 MOI of H1N1 virus (the PR8 strain) and incubated for 24 h. Cells were observed under an Olympus microscope at 40× magnification, and static bright fields were randomly photographed (A). Scale bar, 20 μm. (B and C) LET1 and NL20 cells were infected with the indicated MOI of H1N1 for 24 h or with 2 MOI of H1N1 virus for the indicated lengths of time. Cell lysates were analyzed for the levels of indicated proteins, whereas the conditioned media of LET1 cells (sup.) was analyzed for HMGB1 by western blot. x, non-specific detection of an unknown protein. (D and E) LET1 and NL20 cells were infected with the indicated MOI of H1N1 for 24 h or with 2 MOI of H1N1 virus for the indicated lengths of time. Cell lysates were analyzed for the levels of indicated proteins. (F) J774A.1, iBMM, LET1, and NL20 cells were infected with 5 MOI of H1N1 for 24 h. Cell lysates were analyzed for the levels of NLRP3, NS1, and β-actin.

The mechanisms of IAV-induced pyroptosis have been largely studied in BMDMs.^13^^,^^29^ Wan et al.^30^ recently showed that the H7N9 virus induces pyroptosis in human and murine respiratory epithelial cells via GSDME activation. More recently, Guy et al.^20^ reported that IAV induces pyroptosis in NHBE cells via the PKR-caspase-8-caspase-3-GSDME pathway. Here, we first confirmed the ability of IAV to induce pyroptosis in NL20 and LET1 cells, two respiratory epithelial cell lines. H1N1 virus (PR8) infection at 24 h induced pyroptosis in LET1 and NL20 cells, which was evidenced by the morphological change to the “bubbling” appearance due to osmotic swelling (Figure 1A). H1N1 virus infection dose- and time dependently induced GSDMD and GSDME cleavage in these two cell lines, which generated a truncated N-terminal fragment with a molecular mass around 30 and 34 kDa, respectively (Figures 1B and 1C). HMGB1 released into the conditioned media has been used as a marker to monitor pyroptosis and necroptosis.^31^ Indeed, H1N1 virus infection induced HMGB1 release in LET1 cells in a dose- and time-dependent manner (Figure 1B).

GSDMD can be cleaved and activated by caspase-8 or caspase-1, whereas GSDME can be cleaved and activated by caspase-3.^29^^,^^31^ Here, we tested whether IAV infection induced the activation of these caspases. H1N1 virus infection dose- and time dependently induced caspase-8 cleavage, which produced an 18- and 43-kDa fragment in LET1 and NL20 cells (Figures 1D and 1E). H1N1 virus infection also induced caspase-3 cleavage, which produced a 17- and 19-kDa doublet (Figures 1D and 1E). IAV infection induces caspase-1 activation in macrophages by the inflammasome through the classical NLRP3-ASC-caspase-1 pathway.^13^^,^^32^ NLRP3 was highly expressed in the murine J774A.1 macrophage cell line and in immortalized bone-marrow-derived macrophages (iBMMs) but hardly detectable in LET1 and NL20 cells (Figure 1F). Caspase-1 was expressed at much lower levels in LET1 and NL20 cells than in iBMMs (Figures S1A and S1B). IAV infection induced caspase-1 cleavage in iBMMs but not in LET1 and NL20 cells (Figures S1A and S1B). Caspase-1 knockdown did not impact IAV-induced GSDMD cleavage in LET1 cells (Figure S1C). These observations collectively suggest that IAV-induced pyroptosis is independent of the classical inflammasome pathway in LET1 and NL20 cells.

### IAV-induced pyroptosis relies on caspase-8 and caspase-3

**Figure 2:**
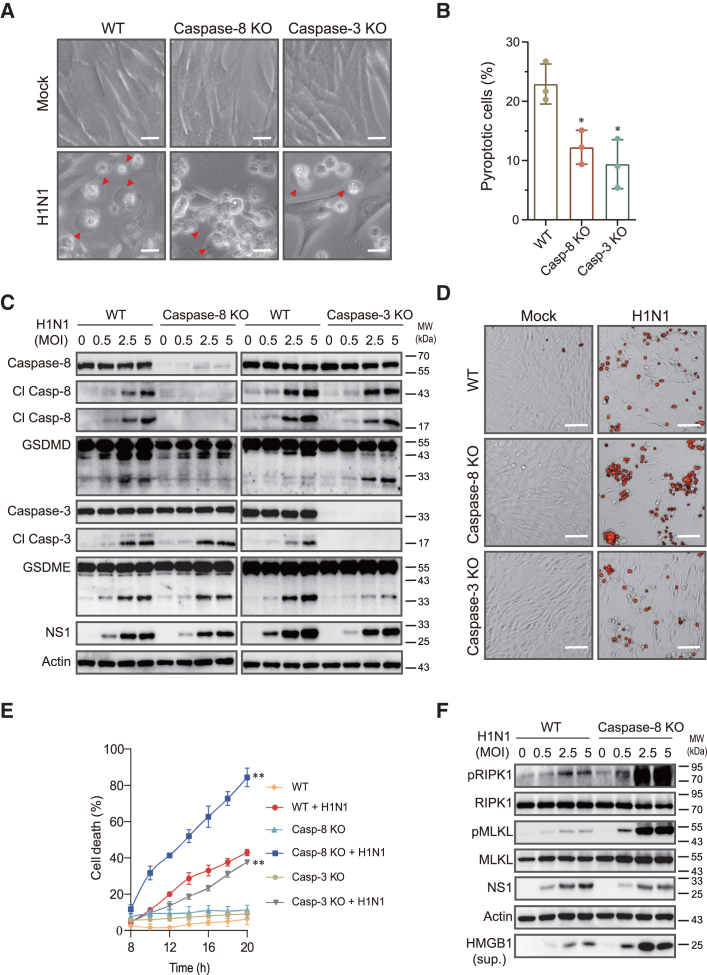
IAV-induced pyroptosis requires caspase-8 and caspase-3 (A and B) Control and caspase-8 or caspase-3 knockout LET1 were infected with 2 MOI of H1N1 for 24 h. Static bright fields were randomly photographed (A). Scale bar, 20 μm. The percentage of pyroptotic cells with a bubbling morphology was calculated and statistically analyzed (B). Data represent the mean ± SD of three independent experiments. Unpaired Student’s t test was used to determine the significant difference between wild-type and caspase-8 or caspase-3-deficient cells. ∗*p* < 0.05. (C and F) Control and caspase-8 or caspase-3 knockout LET1 cells were infected with the indicated MOI of H1N1 and incubated for 24 h. The levels of indicated proteins in cell lysates and HMGB1 in the conditioned media (sup.) were detected by western blot. (D and E) Control and caspase-8 or caspase-3 knockout LET1 cells were infected with 2 MOI of H1N1 virus. Cell death including apoptosis, necroptosis, and pyroptosis was analyzed by PI staining followed by photographing at 14 h post infection (D) or reading in a plate reader at the indicated time points (E). Scale bar, 100 μm. The results represent the mean ± SD of one experiment in triplicate. The experiment was repeated three times with similar results. Repeated measure two-way ANOVA test was used to determine the statistical difference between wild-type and caspase-8 or caspase-3 knockout cells infected with H1N1 viruses. ∗∗*p* < 0.01.

We next tested if caspase-8 and caspase-3 knockout affected IAV-induced pyroptosis. As shown in Figures 2A and 2B, the numbers of pyroptotic cells were much lower in caspase-8 and caspase-3 knockout cells than in the wild-type control cells infected with H1N1 virus. Caspase-8 knockout in LET1 cells abolished IAV-induced GSDMD cleavage but had no effect on GSDME cleavage (Figure 2C). Intriguingly, caspase-8 knockout had little effect on IAV-induced caspase-3 cleavage (Figure 2C). IAV infection activated caspase-9 slightly stronger in caspase-8-deficient LET1 cells than wild-type control cells (Figure S2). This suggests that caspase-3 can be redundantly cleaved and activated by caspase-8 and caspase-9. Caspase-3 knockout had no effect on caspase-8 cleavage but significantly decreased GSDME cleavage in LET1 cells (Figure 2C). Caspase-3 knockout significantly decreased the levels of the 43-kDa GSDMD fragment but increased the levels of the 30-kDa GSDMD protein, which represents the active form of GSDMD (Figure 2C). We then investigated the effects of caspase-8 and caspase-3 knockout on IAV-induced cell death. Propidium iodide (PI) staining revealed that caspase-8 knockout did not decrease but rather increased the number of PI-positive cells and fluorescent signals (Figures 2D and 2E). In contrast, caspase-3 knockout slightly but significantly lowered the number of PI-positive cells and decreased the levels of fluorescent signals in IAV-infected LET1 cells (Figures 2D and 2E).

We next tested if increased cell death in caspase-8 knockout cells was due to increased necroptosis. Caspase-8 knockout dramatically increased RIPK1 and MLKL phosphorylation and the release of HMGB1 into the conditioned media of IAV-infected LET1 cells (Figure 2F). To rule out the possibility that IAV-induced pyroptosis is indirectly mediated through the tumor necrosis factor alpha (TNF-α) receptor (TNFR), we examined the ability of IAV to activate caspases in LET1 cells with TNFR knockdown. TNFR knockdown blocked TNF-α, Smac, Z-Vad-induced MLKL phosphorylation but had no effect on H1N1 virus-induced MLKL phosphorylation and caspase-8/3 cleavage (Figures S3A and S3B), suggesting that IAV-induced pyroptosis is independent of TNF-α.

### Pyroptosis restricts IAV replication

**Figure 3:**
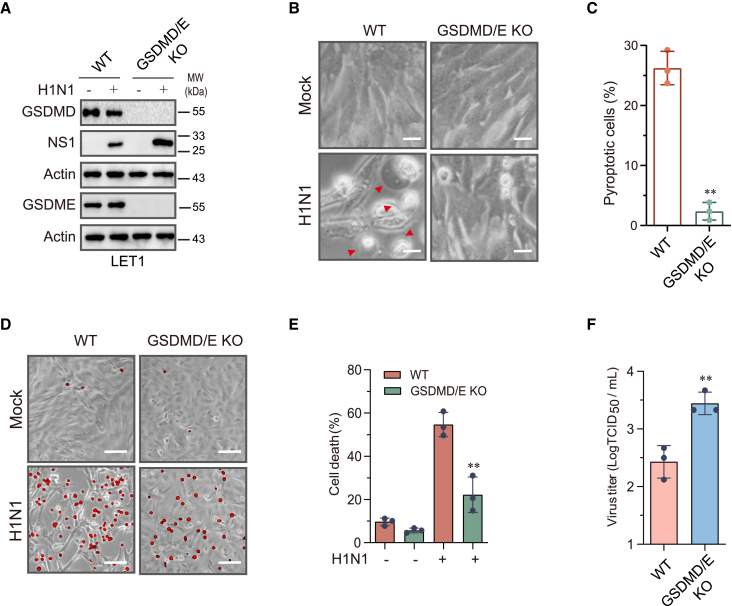
Role of GSDMD and GSDME in cell death and virus replication Control and GSDMD and GSDME double-knockout LET1 were infected with 2 MOI of H1N1 for 24 h. Cell lysates were analyzed for the levels of the indicated proteins by western blot (A). Static bright fields were randomly photographed (B). Scale bar, 20 μm. The percentage of bubbling cells was calculated and statistically analyzed (C). Cell death was analyzed by PI staining followed by photographing at 24 h post infection (D) or reading in a plate reader at the indicated time points (E). Scale bar, 100 μm. The virus titers in the conditioned media were analyzed by measuring the 50% tissue culture infection dose (TCID_50_) values (F). Data represent the mean ± SD of three independent experiments. Unpaired Student’s t test was used to determine the statistical difference between wild-type and GSDMD/E-deficient cells infected with H1N1 viruses. ∗∗*p* < 0.01.

Cell death is considered the first line of defense against virus replication.^13^^,^^29^ We then tested if blocking pyroptosis could enhance IAV replication. Since caspase-8 and caspase-3 are also involved in apoptosis, we knocked out GSDMD and GSDME simultaneously to determine if lack of pyroptosis affected virus replication. GSDMD and GSDME expression was eliminated in clones screened from LET1 cells co-transfected with the LentiCRISPRv2 vector targeting GSDMD and GSDME genes (Figure 3A); H1N1 virus infection did not induce pyroptosis in GSDMD/E-deficient LET1 cells as the cells did not present with the morphologically swelling characteristics of pyroptosis (Figures 3B and 3C). PI staining revealed that GSDMD/E knockout lowered the number of PI-positive cells and fluorescent signals, compared to that in wild-type cells infected with IAV (Figures 3D and 3E). GSDMD/E knockout increased virus replication as evidenced by the elevated levels of the NS1 protein (Figure 3A) and virus titers (Figure 3F). These observations collectively suggest that the blockade of pyroptosis enhances IAV replication.

### IAV-induced pyroptosis requires ZBP1 but not RIPK3

**Figure 4:**
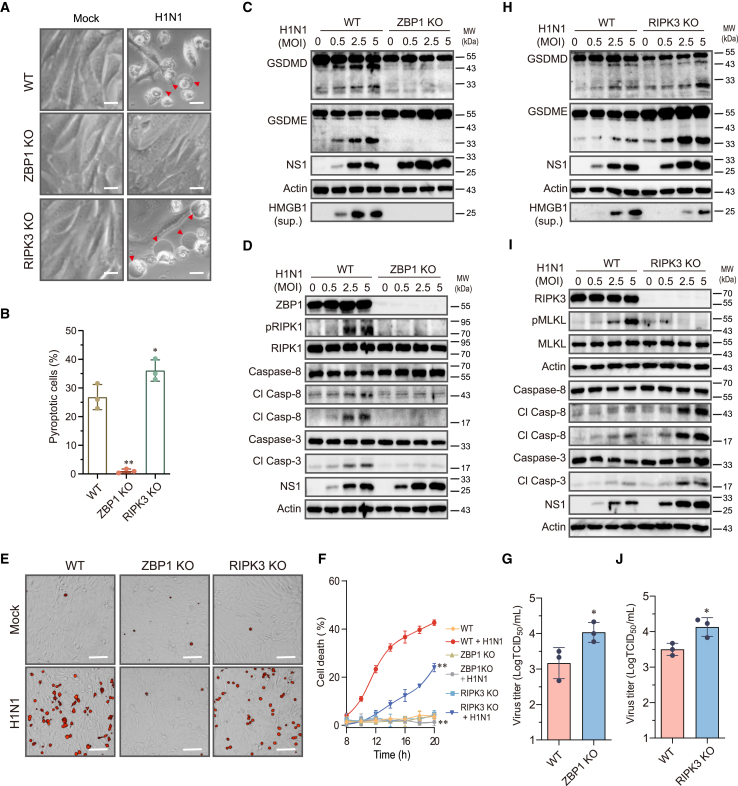
Role of ZBP1 and RIPK3 in IAV-induced pyroptosis Control and ZBP1 or RIPK3 knockout LET1 cells were infected with 2 MOI of H1N1 for 36 h. Static bright fields were randomly photographed (A). Scale bar, 20 μm. The percentage of bubbling cells was calculated and statistically analyzed (B). Data represent the mean ± SD of three independent experiments. ∗*p* < 0.05, ∗∗*p* < 0.01. Control and ZBP1 (C and D) or RIPK3 (H and I) knockout LET1 cells were infected with the indicated MOI of H1N1 viruses and incubated for 24 h. The levels of indicated proteins in cell lysates and HMGB1 in the conditioned media (sup.) were detected by western blot. (E and F) Control and ZBP1 or RIPK3 knockout LET1 cells were infected with 2 MOI of H1N1 virus. Cell death was analyzed by PI staining followed by photographing at 14 h post infection (E) or reading in a plate reader at the indicated time points (F). Scale bar, 100 μm. The results represent the mean ± SD of one experiment in triplicate. The experiment was repeated three times with similar results. Repeated measure two-way ANOVA test was used to determine the statistical difference between wild-type and ZBP1 or RIPK3 knockout cells infected with H1N1 viruses. ∗∗*p* < 0.01. (G and J) Control and ZBP1 or RIPK3 knockout LET1 cells were infected with 2 MOI of H1N1 virus for 24 h. The virus titers in the conditioned media were analyzed by measuring the TCID_50_ values. Data represent the mean ± SD of three independent experiments. ∗*p* < 0.05 (unpaired Student’s t test).

ZBP1 binds RIPK3 to initiate the assembly of the PANoptosome complex, which then activates GSDMD through the NLRP3-ASC-caspase-1 pathway.^14^^,^^33^ Here, we first tested if ZBP1 was also required for IAV-induced pyroptosis in LET1 cells. ZBP1 knockout LET1 cells infected with H1N1 virus for up to 36 h did not undergo pyroptosis (Figures 4A and 4B). In contrast, approximately 26% of wild-type control cells infected with H1N1 virus became pyroptotic (Figure 4B). IAV infection induced GSDMD and GSDME cleavage in wild-type but not ZBP1-deficient LET1 cells (Figure 4C). Consistently, IAV infection induced caspase-8 and caspase-3 cleavage as well as RIPK1 phosphorylation in wild-type cells but not in ZBP1-deficient LET1 cells (Figure 4D). PI staining revealed that ZBP1 knockout blocked IAV-induced cell death (Figures 4E and 4F). Inhibition of cell death by ZBP1 knockout led to increased virus replication, as evidenced by increased levels of the NS1 protein (Figures 4C and 4D) and virus titers (Figure 4G).

RIPK3 has been implicated in playing an important role in inducing caspase-8 activation, apoptosis, and necroptosis in IAV-infected MEFs.^9^^,^^10^ Here, we tested if RIPK3 was also involved in IAV-induced pyroptosis in airway epithelial cells. RIPK3 knockout did not prevent but rather slightly increased the numbers of pyroptotic cells following IAV infection, as evidenced by the bubbling morphology (Figures 4A and 4B). Moreover, RIPK3 knockout enhanced IAV-induced GSDMD and GSDME cleavage, compared to that in wild-type cells (Figure 4H). RIPK3 knockout blocked IAV-induced MLKL phosphorylation but enhanced IAV-induced caspase-8 and caspase-3 cleavage (Figure 4I). Since RIPK3 is required only for IAV-induced necroptosis,^34^ RIPK3 knockout partially decreased cell death in a PI staining assay (Figures 4E and 4F), which led to increased virus replication (Figure 4J). Consistent with this observation, previous studies have shown that IAV infection can still induce cell death in RIPK3-deficient MEFs or primary mouse BMDMs but at a significantly lower level than their wild-type control cells.^10^^,^^35^

### RIPK1 and its kinase activity are required for IAV-induced pyroptosis

**Figure 5:**
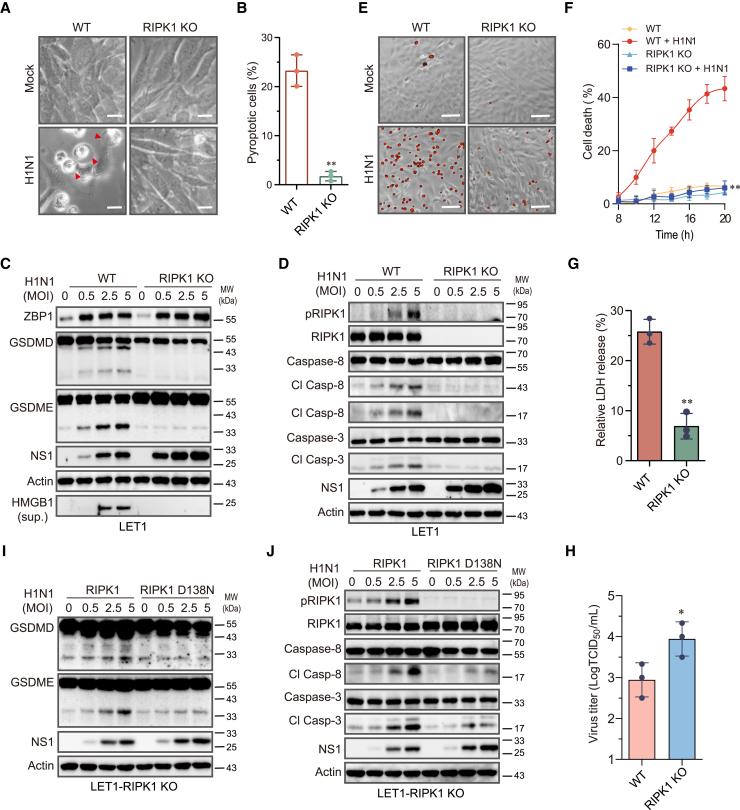
IAV-induced pyroptosis requires RIPK1 and its enzymatic activity (A and B) Control and RIPK1 knockout LET1 were infected with 2 MOI of H1N1 for 24 h. Static bright fields were randomly photographed (A). Scale bar, 20 μm. The percentage of bubbling cells was calculated and statistically analyzed (B). Data represent the mean ± SD of three independent experiments. ∗∗*p* < 0.01. (C and D) Control and RIPK1 knockout LET1 cells were infected with the indicated MOI of H1N1 and incubated for 24 h. Cell lysates were analyzed for the levels of the indicated proteins by western blot. (E–G) Control and RIPK1 knockout LET1 cells were infected with 2 MOI of H1N1 virus. Cell death was analyzed by PI staining followed by photographing at 24 h post infection (E) or reading in a plate reader at the indicated time points (F). Scale bar, 100 μm. The results represent the mean ± SD of one experiment in triplicate. The experiment was repeated three times with similar results. Repeated measure two-way ANOVA test was used to determine the statistical difference between wild-type and RIPK1 knockout cells infected with H1N1 viruses. ∗∗*p* < 0.01. (G) Cell death was analyzed by measuring LDH released into the conditioned media collected at 14 h post infection. Data are the mean ± SD of three independent experiments. (H) Control and RIPK1 knockout LET1 cells were infected with 2 MOI of H1N1 virus for 24 h. The virus titers in the conditioned media were analyzed by measuring the TCID_50_ values. Data represent the mean ± SD of three independent experiments. The unpaired Student’s t test was used to determine the statistical difference between wild-type and RIPK1 knockout cells in (G) and (H). ∗*p* < 0.05; ∗∗*p* < 0.01. (I and J) RIPK1 knockout LET1 cells were transfected with a murine wild-type RIPK1 gene or a kinase-dead (KD) mutant RIPK1 gene (D138N). After incubation for 30 h, the cells were infected with the indicated MOI of H1N1 virus and incubated for 18 h. Cell lysates were analyzed for apoptosis- and pyroptosis-related proteins by western blot.

RIPK1 is dispensable for IAV-induced necroptosis but functions as a scaffold protein to mediate IAV-induced apoptosis in MEFs, whereas its kinase activity is dispensable for IAV-induced caspase-8 activation and apoptosis.^4^^,^^6^ Here, we tested if RIPK1 and its kinase activity were also required for IAV-induced pyroptosis. Almost no pyroptotic cells were present in RIPK1-deficient LET1 cells infected with H1N1 virus (Figures 5A and 5B). RIPK1 knockout abolished IAV-induced GSDMD and GSDME cleavage and HMGB1 release into the conditioned media of IAV-infected LET1 cells (Figure 5C). There was no significant difference in ZBP1 levels between wild-type and RIPK1-deficient LET1 cells (Figure 5C). IAV induced RIPK1^S166^ phosphorylation in a dose-dependent manner in wild-type LET1 cells (Figure 5D). RIPK1 knockout blocked IAV-induced caspase-8 and caspase-3 cleavage (Figure 5D), though the levels of the viral NS1 protein were much higher in RIPK1-deficient cells than that in wild-type LET1 cells. PI staining (Figures 5E and 5F) and LDH release assay (Figure 5G) revealed that RIPK1 knockout prevented IAV-induced cell death. Moreover, RIPK1 knockout in LET 1 cells also increased virus titers in the conditioned media (Figure 5H). We next tested whether RIPK1 activity was required for IAV-induced pyroptosis. Re-expression of wild-type RIPK1 but not kinase-dead RIPK1 (D138N) in RIPK1 knockout cells restored the ability of IAV to cleave GSDMD, GSDME, caspase-8, and caspase-3 (Figures 5I and 5J), suggesting that RIPK1 activity is required for IAV-induced caspase-8 activation and pyroptosis.

### TRIF suppresses IAV-induced pyroptosis by TAK1

**Figure 6:**
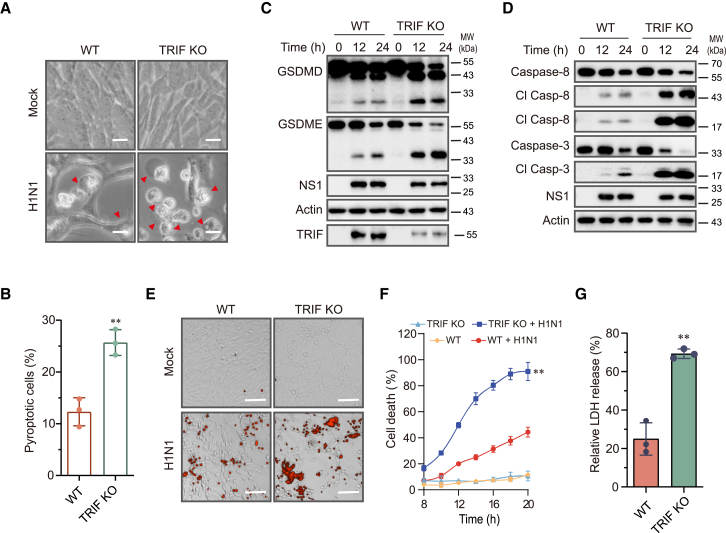
TRIF knockout enhances IAV-induced pyroptosis (A and B) Control and TRIF knockout LET1 infected with 2 MOI of H1N1 were incubated for 14 h. Static bright fields were randomly photographed (A). Scale bar, 20 μm. The percentage of bubbling cells was calculated and statistically analyzed (B). Data represent the mean ± SD of three independent experiments. Unpaired Student’s t test was used to determine the significant difference between wild-type and TRIF knockout cells. ∗∗*p* < 0.01. (C and D) Control and TRIF knockout LET1 cells were infected with 2 MOI of H1N1 virus for the indicated lengths of time. The levels of indicated proteins in cell lysates were detected by western blot. (E–G) Control and TRIF knockout LET1 cells were infected with 2 MOI of H1N1 virus. Cell death was analyzed by PI staining followed by photographing at 14 h post infection (E) or reading in a plate reader at the indicated time points (F). Scale bar, 100 μm. The results represent the mean ± SD of one experiment in triplicate. The experiment was repeated three times with similar results. Repeated measure two-way ANOVA test was used to determine the statistical difference between wild-type and TRIF knockout cells infected with H1N1 viruses. ∗∗*p* < 0.01. (G) Cell death was analyzed by measuring LDH released into the conditioned media collected at 14 h post infection. Data represent the mean ± SD of three independent experiments. Unpaired Student’s t test was used to determine the significant difference between wild-type and TRIF knockout cells. ∗∗*p* < 0.01.

A couple of recent studies indicate that TLR4 and TLR3 activation induces pyroptosis through the TRIFosome.^22^^,^^23^ TRIF knockout blocked LPS plus 5Z-oxzeneonal (5Z)-induced cell death in BMDMs.^22^^,^^23^ Here, we examined the role of TRIF in IAV-induced pyroptosis. The number of pyroptotic cells was significantly increased in TRIF knockout LET1 cells than in wild-type control cells (Figures 6A and 6B). TRIF was not expressed in LET1 cells but was readily induced following IAV infection (Figure 6C). TRIF knockout did not block but rather enhanced IAV-induced cleavage of GSDMD, GSDME, caspase-8, and caspase-3 (Figures 6C and 6D). TRIF knockout was incomplete since low levels of TRIF were detected at 12 and 24 h post virus infection (Figure 6C). PI staining (Figures 6E and 6F) and LDH release assay (Figure 6G) revealed that TRIF knockdown enhanced IAV-induced cell death.

**Figure 7:**
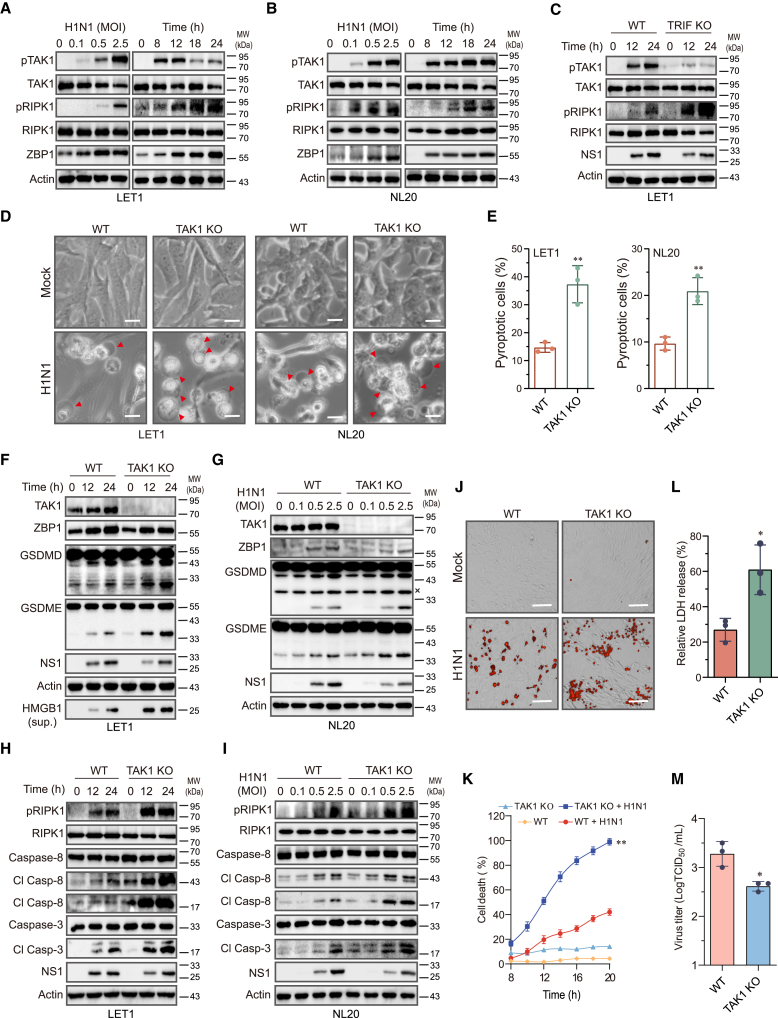
TAK1 knockout activates RIPK1 and promotes pyroptosis (A and B) LET1 and NL20 cells were infected with the indicated MOI of H1N1 for 24 h or with 2 MOI of H1N1 virus for the indicated lengths of time. (C) Control and TRIF knockout LET1 cells were infected with 2 MOI of H1N1 virus for the indicated lengths of time. Cell lysates were analyzed for the levels of indicated proteins by western blot. (D and E) Control and TAK1 knockout LET1 or NL20 cells were infected with 2 MOI of H1N1 for 14 h. Static bright fields were randomly photographed (D). Scale bar, 20 μm. The percentage of bubbling cells was calculated and statistically analyzed (E). Data represent the mean ± SD of three independent experiments. Unpaired Student’s t test was used to determine the significant difference between wild-type and TAK1 knockout cells. ∗∗*p* < 0.01. (F–I) Wild-type and TAK1 knockout LET1 and NL20 cells were infected with the indicated MOI of H1N1 for 24 h or with 2 MOI of H1N1 virus for the indicated lengths of time. The levels of indicated proteins in cell lysates and HMGB1 in the conditioned media (sup.) were detected by western blot. (J–L) Control and TAK1 knockout LET1 cells were infected with 2 MOI of H1N1 virus. Cell death was analyzed by PI staining followed by photographing at 14 h post infection (J) or reading in a plate reader at the indicated time points (K). Scale bar, 100 μm. Data represent the mean ± SD of one experiment in triplicate. Repeated measure two-way ANOVA test was used to determine the statistical difference between wild-type and TAK1 knockout cells infected with H1N1 viruses. The experiment was repeated three times with similar results. ∗∗*p* < 0.01. (L) Cell death was analyzed by measuring LDH released into the conditioned media collected at 14 h post infection. (M) Control and TAK1 knockout LET1 cells were infected with 5 MOI of H1N1 virus for 14 h. The virus titers in the conditioned media were analyzed by measuring the TCID_50_ values. Data in (L) and (M) represent the mean ± SD of three independent experiments. Unpaired Student’s t test was used to determine the significant difference between wild-type and TAK1 knockout cells. ∗*p* < 0.05.

TAK1 is a key serine/threonine kinase downstream of TLR3 and plays an important role in blocking TNF-α- and LPS-induced cell death.^24^^,^^36^ We speculated that TRIF may activate TAK1 to block IAV-induced pyroptosis. Indeed, IAV induced TAK1 and RIPK1 phosphorylation and ZBP1 expression in a time- and -dose-dependent manner in NL20 and LET1 cells (Figures 7A and 7B). TRIF knockdown in LET1 cells blocked IAV-induced TAK1 phosphorylation but dramatically increased RIPK1^S166^ phosphorylation (Figure 7C). TAK1 knockout significantly increased the number of dead cells with the characteristics of pyroptosis in IAV-infected LET1 and NL20 cells (Figures 7D and 7E). TAK1 knockout enhanced IAV-induced GSDMD and GSDME cleavage in LET1 (Figure 7F) and NL20 (Figure 7G) cells. Consistently, TAK1 knockout enhanced IAV-induced RIPK1^S166^ phosphorylation as well as caspase-8 and caspase-3 cleavage in LET1 (Figure 7H) and NL20 (Figure 7I) cells. Of note, the effect of TAK1 knockout on enhancing caspase-8/3 and GSDMD/E activation appeared to be much stronger in LET1 cells than in NL20 cells (Figures 7F–7I). PI staining (Figures 7J and 7K) and LDH release assay (Figure 7L) revealed that TAK1 knockdown enhanced IAV-induced cell death in LET1 cells but modestly lowered virus titers (Figure 7M).

### 5Z treatment enhances cell death in IAV-infected lungs

**Figure 8:**
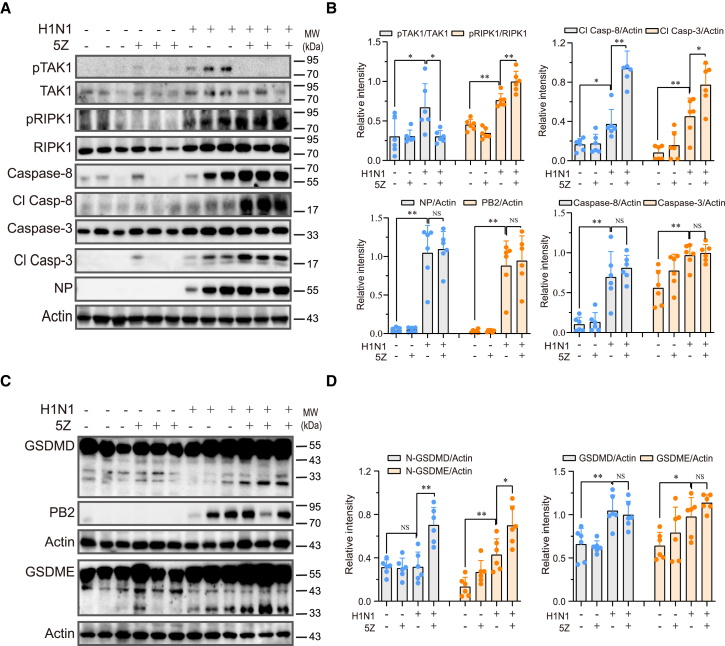
5Z enhances IAV-induced pyroptosis *in vivo* (A–D) Female C57BL/6 mice (6 mice/group) were divided into 4 groups. The mice were either mock-infected or infected with H1N1 viruses at a dosage of 1,000 pfu/mouse. One day after infection, the mice were treated daily with vehicle or 5Z at a dose of 2 mg/kg bodyweight for two consecutive days. On the third day post infection, the mice received a final dose of 5Z at 8 h prior to sacrifice. Lung tissue lysates were prepared and analyzed for the levels of indicated proteins by western blot. The band densities from 6 mice per group were analyzed using NIH ImageJ software and normalized by the arbitrary units of their total protein bands or β-actin levels. ^∗^*p* < 0.05; ^∗∗^*p* < 0.01.

Finally, we investigated the effect of TAK1 inhibition on pyroptosis *in vivo* in the lung tissues of IAV-infected mice. As shown in Figures 8A and 8B, IAV infection significantly increased TAK1 and RIPK1 phosphorylation in the lysates of lung tissues, which was inhibited by 5Z treatment (2 mg/kg body weight) for 2 days (Figures 8A and 8B). IAV infection significantly increased the expression of caspase-8 and caspase-3 and induced their cleavage (Figures 8A and 8B). 5Z treatment enhanced the cleavage of caspase-8 and caspase-3 proteins (Figures 8A and 8B) in the lung tissues of H1N1 virus-infected mice. Additionally, 5Z treatment promoted the cleavage of GSDMD and GSDME (Figures 8C and 8D). 5Z treatment had no effect on the levels of NP and PB2 proteins (Figures 8A and 8C). These findings indicate that 5Z treatment activates RIPK1 *in vivo* and enhances IAV-induced pyroptosis in the lungs of IAV-infected mice.

## Discussion

Pyroptosis is a form of programmed cell death that allows intracellular substances such as HMGB1 to be released into the extracellular space and alert the immune system and elicit antiviral immunity.^13^^,^^29^ Our present study provides evidence that IAV-induced pyroptosis in two respiratory epithelial cell lines is mediated by activating caspase-8 and its downstream caspase-3 to cleave GSDMD and GSDME, respectively. Caspase-8 is activated through the ZBP1-RIPK1-FADD-caspase-8 complex (graphical abstract). TAK1 activation via TRIF suppresses RIPK1 activation and attenuates IAV-induced caspase-8 activation and pyroptosis. Our study provides mechanistic insights into how IAV induces pyroptosis in airway epithelial cells and how influenza virus evolves a strategy to repress pyroptosis.

IAV infection induces pyroptosis in macrophages by activating the NLRP3 inflammasome and caspase-1, which then cleave and activate GSDMD and IL-1β.^18^ Activated GSDMD oligomerizes and anchors into the plasma membrane to form pores through which mature IL-1β is released into the extracellular space. Zheng et al.^14^^,^^37^ reported that caspase-6 facilitates ZBP1 binding to RIPK1 and stabilizes the PANoptosome that is required for NLRP3 activation. However, how the ZBP1-RIPK3-RIPK1-FADD-caspase-8 complex activates NLRP3 remained unclear. Lei et al.^19^ recently reported that the NLRP3-mediated inflammatory cell death in the early stage of IAV infection is activated by K^+^ efflux through MLKL pores. In addition to macrophages, IAV also induces pyroptosis in respiratory epithelial cells. For example, IAV infection triggers apoptosis and pyroptosis of respiratory epithelial cells in a mutually exclusive manner^38^; H5N1 virus infection induces pyroptosis in alveolar macrophages and epithelial cells in macaques by activating GSDMD^39^; and H7N9 virus induces pyroptosis in lung alveolar epithelial cells through GSDME activation.^30^ Recently, Guy et al.^20^ reported that IAV infection induces pyroptosis in NHBE cells by activating GSDME through caspase-3. Our present study shows that IAV infection induced pyroptosis in NLRP3-negative LET1 and NL20 cells by caspase-8-activated GSDMD and caspase-3-activated GSDME but not by caspase-1 since caspase-1 was not activated in IAV-infected LET1 and NL20 cells; caspase-1 knockdown did not affect GSDMD activation in LET1 cells (Figure S1C).

We then investigated the mechanisms of caspase-8 activation in IAV-infected epithelial cells. We found that ZBP1 and RIPK1 knockout abolished or reduced caspase-8/3 and GSDMD/E activation in IAV-infected LET1 cells, whereas RIPK3 knockout enhanced caspase-8/3 and GSDMD/E activation. These observations suggest that IAV infection activates caspase-8 by the ZBP1-RIPK1-FADD-caspase-8/3 pathway (graphical abstract). We further show that the enzymatic activity of RIPK1 was required for IAV-induced caspase-8 activation. In contrast, Lei et al.^19^ reported that IAV infection activates caspase-8 and induces macrophage pyroptosis in a RIPK1-independent but RIPK3-dependent manner. Balachandran and colleagues reported that IAV infection activates caspase-8 in a RIPK3-dependent but RIPK3 and RIPK1 activity-independent fashion in MEFs.^9^^,^^10^ They proposed that IAV activates caspase-8 by the ZBP1-RIPK3-RIPK1-FADD-caspase-8 pathway in MEFs. This pathway contrasts with ours (graphical abstract) in this and prior studies, where IAV infection activates caspase-8 in a RIPK1 activity-dependent but RIPK3-independent manner.^34^ While we and others have shown that ZBP1 and RIPK1 play a crucial role in activating caspase-8 in murine airway epithelial cells, MEFs, and macrophages.^32^ Guy et al.^20^ recently reported that PKR is required for IAV-induced caspase-8 activation in primary normal human bronchial epithelial cells. We did not fully investigate the role of PKR in mediating IAV-induced caspase activation and pyroptosis in our human NL20 cell line. We also were unsuccessful in knocking out ZBP1 in NL20 cells to confirm its role in IAV-induced cell death. Therefore, whether IAV induces caspase activation and pyroptosis in human cells via a mechanism identical to or overlapping with that in murine cells remains elusive.

IL-1 and the NLRP3 inflammasome play a critical role in the antiviral immune response against influenza virus, especially those zoonotic subtypes such as H5N1 and H7N9 viruses.^13^^,^^29^^,^^33^ However, excessive inflammatory cytokines caused by H7N9 virus trigger a cytokine storm that is detrimental to hosts.^30^ Mice deficient of GSDME display the lower levels of pyroptosis in lung alveolar epithelial cells than wild-type mice infected with H7N9 virus.^30^ It appears that influenza viruses have evolved some strategies to limit pyroptosis to benefit their replication. For example, PB1-F2, a small accessory protein that is predominantly expressed in the zoonotic H5N1 and H7N9 viruses as well as contemporary human H3N2 viruses, binds NLRP3 and suppresses inflammasome and pyroptosis in human THP-1 macrophages.^40^ The NS1 protein of 2009 pandemic IAV inhibits ASC ubiquitination and NLRP3 inflammasome-activated IL-1β production.^41^ Our present study shows that TAK1, which was readily activated by IAV infection, downregulated H1N1 virus-induced pyroptosis by suppressing RIPK1 activation. Inhibition of RIPK1 activity by Nec-1s blocked IAV-induced caspase-8 activation and cell death (Figures S4A and S4B). TAK1 activates p38-MK2 to phosphorylate RIPK1 at Ser-321^42^^,^^43^ or activate IKK to phosphorylate RIPK1 at Ser-25 to suppress its activation.^25^^,^^44^^,^^45^ Our recent study showed that TAK1 activates IKK to suppress RIPK1 and caspase-8 activation in IAV-infected cells.^34^

TRIF is an adaptor protein of the TLR signaling pathway that primarily functions to activate TAK1 and several downstream kinases such as p38, ERK, JNK, and IKK. However, TRIF can also trigger LPS/5Z-mediated cell death in macrophages through TRIFosome, a complex that contains TRIF, ZBP1, RIPK1, FADD, and caspase-8.^22^^,^^23^ TRIF knockout blocks LPS/5Z-induced caspase-8 activation and pyroptosis.^23^ We also investigated the role of TRIF in IAV-induced cell death. TRIF knockout did not inhibit but rather enhanced IAV-induced caspase-8 activation and pyroptosis. Further experiments revealed that TRIF deficiency-enhanced cell death was mediated by the suppression of TAK1 activation. 5Z treatment led to increased caspase-8 and caspase-3 activation as well as increased GSDMD and GSDME cleavage *in vivo* (Figure 8). These observations collectively suggest that IAV activates TAK1 via TRIF to repress RIPK1 activation, leading to decreased caspase and GSDM activation as well as decreased pyroptosis.

Cell death is a “double-edged sword” in viral pathogenesis.^4^^,^^31^ Host cells commit “suicide” to restrict virus replication and spread to adjacent cells, and to alert the immune system to eliminate virus-infected cells.^5^^,^^46^ However, excessive cell death, particularly necroptosis and pyroptosis, may trigger an inflammatory cytokine “storm” that is ultimately detrimental to hosts.^5^^,^^46^ Gautam et al.^47^ recently reported that inhibition of necroptosis by UH15-38, a RIPK3-specific inhibitor, or by MLKL deficiency, blocks H1N1 virus (PR8)-induced lung injury in a mouse model of severe influenza. In contrast, ZBP1 deficiency, which prevents cell death but promotes virus replication, does not protect mice from lethal H1N1 virus infections.^47^ Our recent study showed that RIPK1 is a kinase immediately downstream of ZBP1 and is required for both IAV-induced apoptosis and necroptosis,^34^ suggesting that ZBP1 and RIPK1 deficiency will have a similar impact on IAV infection. Indeed, mice lacking RIPK1 activity are equally susceptible to IAV infection as do wild-type mice.^47^ Furthermore, treatment with the RIPK1 inhibitor Nec-1 does not protect mice from IAV infection.^48^ Our present study shows that IAV infection induced pyroptosis by activating the ZBP1-RIPK1-caspase-8/3-GSDMD/E pathway (graphical abstract). Mice deficient in GSDME survive the lethal infection of the highly pathogenic H7N9 virus.^30^ GSDMD deficiency also abrogates IAV-induced inflammatory responses in the lungs of IAV (HKx31) virus-infected mice and prolongs their survival.^49^ These findings suggest that targeting necroptosis or pyroptosis alone is sufficient to prevent severe influenza.^30^ Interestingly, two recent studies showed that targeting caspase or intrinsic apoptosis ameliorates viral pathogenesis in the mouse models of Middle East respiratory syndrome coronavirus, severe acute respiratory syndrome coronavirus (SARS-CoV), SARS-CoV-2, and coxsackievirus infections.^50^^,^^51^ It remains unclear if targeting apoptosis alone could also offer protection against IAV infection. Our recent and current studies showed that treatment with the TAK1 inhibitor 5Z aggravated all three forms of cell death in the lungs of IAV-infected mice.^34^ 5Z treatment does not shorten but slightly prolong the survival of IAV-infected mice.^34^^,^^52^ This is likely because TAK1 is a multifunctional kinase that can promote inflammation by activating nuclear factor κB and JNK^24^^,^^53^ and by disrupting the intercellular tight junctions.^52^ In addition, TAK1 can promote autophagy to enhance virus replication.^54^ Therefore, TAK1 inhibition has a very complicated effect on lung inflammation and animal survival.

In summary, our study provides evidence that IAV induces pyroptosis through caspase-8-activated GSDMD and caspase-3-activated GSDME in airway epithelial cells; IAV infections activate caspase-8 via the ZBP1-RIPK1-FADD-caspase-8 complex; and TAK1 activation via TRIF attenuates IAV-induced pyroptosis by blocking RIPK1 activation. Our study provides mechanistic insights into how IAV infection manipulates pyroptosis in respiratory epithelial cells.

### Limitations of the study

Our study has several limitations. (1) TAK1 regulation of RIPK1 activity in IAV-induced cell death in respiratory epithelial cells has not been confirmed *in vivo* in tissue-specific conditional knockout mice. (2) While we and others have shown that ZBP1 plays a dominant role in activating caspase-8/3 and GSDMD/E in murine cells,^32^ how much the ZBP1-RIPK1 axis and PKR or other pathways such as PERK-driven endoplasmic reticulum (ER) stress contribute to caspase activation and pyroptosis in human cells remains unknown. (3) We and others have demonstrated that caspase-3 is activated in alveolar epithelial cells of IAV-infected mice^30^^,^^34^; it is highly likely that GSDMD and GSDME are activated in bronchial and alveolar epithelial cells, but this needs to be verified by direct evidence. (4) NLRP3 was not detected in both LET1 and NL20 cells, whereas caspase-1 was expressed at low levels and not activated in these two cell lines. If this is also the case *in vivo*, the peripatetic cytokines such as IL-1β and IL-18 will be cleaved only by caspase-1 produced by macrophages or other inflammatory cells. However, other “danger” signals such as ATP and HMGB1 released by pyroptotic cells do have a role in alerting the immune system and in adaptive immunity. (5) The magnitude of pyroptosis induced by different IAV subtypes is variable. PR8, an H1N1 strain that has been adapted in mice, was used in our study. Whether the observations made *in vivo* with this H1N1 virus could be extended to other subtypes remains unclear.

## Resource availability

### Lead contact

Further information and requests for resources and reagents should be directed to and will be fulfilled by the lead contact, Xiulong Xu (xxl@yzu.edu.cn).

### Materials availability

Plasmids and the viruses used in this study can be obtained from the lead contact after the permission of original distributors.

### Data and code availability

• All data reported in this paper will be shared by the lead contact upon request.
• This paper does not report original code.
• Any additional information required to reanalyze the data reported in this paper is available from the lead contact upon request.

## Acknowledgments

This work was supported in part by the National Key Research and Development Project of China (2021YFD1800202) (X.W.) and the 10.13039/501100012246Priority Academic Program Development of Jiangsu Higher Education Institutions (X.X.). We thank Dr. Jiahuai Han for kindly providing wild-type and KD-RIPK1 plasmids, Dr. Liqian Zhu (College of Veterinary Medicine, Yangzhou University) for kindly providing the H1N1 (PR8) virus, BEI Resources for providing the LET1 cell line, Prof. Zhenfan Jiang (Peking University) for kindly providing iBMM cells, and BioRender (biorender.com) for providing items for drawing graphic abstract.

## Author contributions

Conceptualization, X.X.; methodology, Y.S., X.X., W.L., P.L., and P.Z.; investigation, Y.S., H.Y., and Z.Z.; supervision, J.S.; writing – original draft, Y.S.; writing – review and editing, X.X.; funding acquisition, X.X. and X.W.; resources, X.X., X.L., and X.W.

## Declaration of interests

The authors declare no competing interests.

## STAR★Methods

### Key resources table

**Table:** 

REAGENT or RESOURCE	SOURCE	IDENTIFIER
**Antibodies**		

Rabbit monoclonal anti-MLKL (phospho-S345)	Abcam	Cat#ab196436; RRID:AB_2687465
Rabbit polyclonal anti-TNFR1	Abcam	Cat#223352; RRID:AB_1267363
Rabbit monoclonal anti-GSDME	Abcam	Cat#215191; RRID:AB_2737000
Rabbit monoclonal anti-GSDMD	Abcam	Cat#209845; RRID:AB_2783550
Rabbit monoclonal anti-RIPK3 (phospho-T231/S232)	Abcam	Cat#222320;
Rabbit monoclonal anti-MLKL (D6W1K)	Cell Signaling Technology	Cat#37705; RRID:AB_2799118
Rabbit monoclonal anti-RIPK3 (D4G2A)	Cell Signaling Technology	Cat#95702; RRID:AB_2721823
Rabbit monoclonal anti-RIPK1 (phospho-S166)	Cell Signaling Technology	Cat#53286; RRID:AB_2925183
Rabbit monoclonal anti-RIPK1 (D94C12)	Cell Signaling Technology	Cat#3493; RRID:AB_2305314
Rabbit monoclonal anti-Caspase-8	Cell Signaling Technology	Cat#4790; RRID:AB_10545768
Rabbit monoclonal anti-Mouse Cleaved Caspase-8 (Asp387)	Cell Signaling Technology	Cat#8592; RRID:AB_10891784
Rabbit monoclonal anti-Human Cleaved Caspase-8 (Asp391)	Cell Signaling Technology	Cat#9496; RRID:AB_561381
Rabbit monoclonal anti-Caspase-3 (D3R6Y)	Cell Signaling Technology	Cat#14220; RRID:AB_2798429
Rabbit monoclonal anti-Cleaved caspase-3	Cell Signaling Technology	Cat#9664; RRID:AB_2070042
Rabbit monoclonal anti-Phospho-TAK1 (Thr187)	Cell Signaling Technology	Cat#4536; RRID:AB_330493
Rabbit monoclonal anti-TAK1	Cell Signaling Technology	Cat#4505; RRID:AB_490858
Rabbit monoclonal anti-Gasdermin D (E8G3F)	Cell Signaling Technology	Cat#97558; RRID:AB_2864253
Rabbit monoclonal anti-Caspase-1 (E8G3F)	Cell Signaling Technology	Cat#3866; RRID:AB_2069051
Rabbit monoclonal anti-NLRP3 (D4D8T)	Cell Signaling Technology	Cat#15101; RRID:AB_2722591
Rabbit polyclonal anti-Influenza A virus PB2	GeneTex	Cat#GTX125926; RRID: AB_11162999
Rabbit monoclonal anti-Influenza A virus NP	GeneTex	Cat#GTX125989; RRID:AB_11168364
Mouse monoclonal anti-Influenza A NS1	Santa Cruz Biotechnology	Cat#sc-130568; RRID:AB_2011757
Mouse monoclona anti-TICAM-1	Santa Cruz Biotechnology	Cat#sc-514384; RRID:AB_2819024
Mouse monoclonal anti-β-Actin	Santa Cruz Biotechnology	Cat#sc-47778; RRID:AB_626632
Mouse monoclona anti-ZBP1	AdipoGen	Cat#AG-20B-0010; RRID:AB_2490191
Rabbit monoclonal anti-HMGB1	ABclonal Technology	Cat#A19529;

**Bacterial and virus strains**		

H1N1 influenza virus strain A/PR/8/1934 (PR8)	Dr. Liqian Zhu (Yangzhou University, China)	N/A

**Chemicals, peptides, and recombinant proteins**		

Necrostatin 2 racemate (Nec-1s)	Selleck	Cat#S8641
(5Z)-7-Oxozeaenol	Cayman Chmical	Cat#17459
TurboFect Transfection Reagent	Thermo Scientific	Cat#R0531
Propidium iodide	Beyotime	Cat#ST511
T4 DNA ligase	Thermo Scientific	Cat#15224090
Bmsb I restriction enzyme	New England Biolabs	Cat#R0739S
Kpn I restriction enzyme	Thermo Scientific	Cat#FD0524
Nhe I restriction enzyme	Thermo Scientific	Cat#FD0974
DMEM	GIBCO	Cat#12100-046
Ham's F-12	GIBCO	Cat#21700-075
2×EasyTaq PCR SuperMix (+dye )	TransGen Biotech	Cat# AS111-11
OPTI-MEM® medium	GIBCO	Cat# 31985-070
Dimethyl sulfoxide (DMSO)	Sigma	Cat# D2650
Puromycin	Solarbio	P8230
TurboFect Transfection Reagent	Thermo Scientific	Cat#00596562

**Critical commercial assays**		

Seamless Cloning and Assembly Kit	Transgen	Cat# CU101-01
Cytotoxicity Detection Kit (LDH)	Sigma-Aldrich	Cat#11644793001

**Experimental models: Cell lines**		

LET1	BEI Resources	Cat#NR-42941; RRID:CVCL_A7VS
NL20	ATCC	Cat#CRL-2503; RRID:CVCL_3756
J774.1	ATCC	Cat#TIB-67; RRID:CVCL_0358
Immortalized bone marrow macrophages	Prof. Zhenfan Jiang (Peking University, China)	N/A

**Experimental models: Organisms/strains**		

C57BL/6J mice	Charles River	N/A

**Oligonucleotides**		

PCR primer for pCAGGS-mRIPK1 (See Table S1)	This paper	N/A
PCR primer for pCAGGS-mRIPK1 D138N (See Table S1)	This paper	N/A
Mouse TAK1 gRNA target sequence (See Table S2)	This paper	N/A
Human TAK1 gRNA target sequence (See Table S2)	This paper	N/A
Mouse TRIF gRNA target sequence (See Table S2)	This paper	N/A
Mouse RIPK1 gRNA target sequence (See Table S2)	This paper	N/A
Mouse ZBP1 gRNA target sequence (See Table S2)	This paper	N/A
Mouse RIPK3 gRNA target sequence (See Table S2)	This paper	N/A
Mouse Caspase-3 gRNA target sequence (See Table S2)	This paper	N/A
Mouse Caspase-8 gRNA target sequence (See Table S2)	This paper	N/A
Mouse TNFR gRNA target sequence (See Table S2)	This paper	N/A
Mouse GSDMD gRNA target sequence (See Table S2)	This paper	N/A
Mouse GSDME gRNA target sequence (See Table S2)	This paper	N/A

**Recombinant DNA**		

pCAGGS-mRIPK1 plasmid	This paper	N/A
pCAGGS-mRIPK1 D138N plasmid	This paper	N/A
pBOB-mRIPK1 plasmid	Prof. Jiahuai Han (Xiamen University, China)	N/A
pBOB-mRIPK1 D138N plasmid	Prof. Jiahuai Han (Xiamen University, China)	N/A

**Software and algorithms**		

CRISPR Guide RNA Design Tool	Benchling	https://www.benchling.com/crisp
GraphPad software 8.0.2	GraphPad Prism	https://www.graphpad.com/scientific-software/prism
ImageJ	NIH, Bethesda, MD, USA	https://imagej.nih.gov/ij/
Adobe Photoshop CC 2018	Adobe	https://www.adobe.com/

### Experimental model and study participant details

#### Cell culture and virus

NL20 (a human non-tumoral bronchial epithelial cell line) and J774.1 (a murine macrophage cell line) cells were purchased from the American Tissue Culture Collection (Manassas, VA). LET1 cells (a murine lung epithelial type I cell line) were kindly provided by BEI Resources (Manassas, VA). Immortalized bone marrow macrophages (iBMM) were kindely provided by Prof. Zhenfan Jiang (Peking University). Upon their arrival, all cell lines were expanded, reserved in liquid nitrogen, and used within 30 passages. All cell lines were tested negative for mycoplasma contamination by PCR. NL20 cells were grown in ham's F12 medium with 1.5 g/L sodium bicarbonate, 2.7 g/L glucose, 2.0 mM L-glutamine, 0.1 Mm nonessential amino acids, 0.005 mg/ml insulin, 10 ng/ml epidermal growth factor, 0.001 mg/ml transferrin, 500 ng/ml hydrocortisone and 4% fetal bovine serum. LET1 cells were grown in DMEM containing 10% fetal bovine serum (FBS). A/PR8/34 (H1N1) virus was kindly provided by Dr. Liqian Zhu (College of Veterinary Medicine, Yangzhou University). The virus stock was diluted at 1:10,000 in PBS and then inoculated into the allantoic cavity of 10-day-old specific-pathogen-free embryonated chicken eggs (200 μL/egg). Embryos deceased between 60-72 h post infection were preserved at 4°C for 4 h. The allantoic fluid were then collected, centrifuged, aliquoted, and stored at -80°C for use. Virus titers were determined through a 10-fold serial dilution technique, ranging from 10^1^ to 10^9^ dilutions. MDCK cells were then infected with dilutions ranging from 10^1^ to 10^9^. The 50% tissue culture infection dose (TCID50/100 μl) values were calculated using the well-established Reed and Muench method.

#### Virus titration

Control and gene knockout LET1 seeded in 24-well plates were infected with 2 MOI of H1N1 for the indicated time. Conditioned media were collected and analyzed for TCID_50_ values in MDCK cells. Data in bar graphs represents the mean ± SD of three independent experiments.

#### Animals

Animal use was approved by the Institutional Animal Care and Use Committee of Yangzhou University (Approval number 202102006; date of approval: February 5, 2021) and carried out in accordance with the Guide for the Care and Use of Laboratory Animals by the National Research Council. C57BL/6J mice (female, 6-8 weeks) were purchased from Charles River (Beijing, China). All mice were housed in ventilated cages under a 12-hour light/dark cycle at an ambient temperature of 23°C. They were provided with *ad libitum* access to a normal chow diet (NCD). A stock solution of 5Z was prepared by dissolving it in dimethyl sulfoxide (DMSO) at a concentration of 20 mg/ml and subsequently diluted in PBS prior to use. Before virus infection, mice were deprived of water for 6 hours. Anesthesia was induced by intraperitoneal injection of sodium pentobarbital (100 mg/kg body weight) prior to intranasal infection with H1N1 virus (1000 PFU/mouse in 50 μl of PBS). The following day, mice were treated daily with either the vehicle or 5Z (2 mg/kg body weight) for 2 consecutive days. The dosage of 5Z was determined according to our previous study in IAV.^52^ On the third day post-infection, mice received a final dose of 5Z administered 8 hours before sacrifice. Euthanasia was performed by cervical dislocation. One part of the lung tissue was lysed in NP-40 lysis buffer (at a weight/volume ratio of 1:15) and homogenized. The resulting cell lysates were then subjected to Western blot analysis to detect the proteins of interest.

### Method details

#### Pyroptosis analysis

LET1 and NL20 cells seeded in 24-well plates were left uninfected or infected with H1N1 viruses (2 MOI) for the indicated lengths of time. Static bright fields were randomly photographed under an Olympus microscope at 40x magnification. Cells with and without a bubbling morphology in each image (approximately 70-90 cells/field) were counted. The percentage of cells undergoing pyroptosis (with bubbling characteristics) was calculated by dividing the number of pyroptotic cells with the number of all cells in each field. The mean percentage of pyroptotic cells in five randomly selected fields were calculated. The mean percentage from three independent experiments were then pooled and statistically analyzed for significant differences between wild-type and gene knockout cells infected with IAV.

#### Western blot

Cells cultured in 12-well plates were harvested and lysed using NP-40 lysis buffer (50 mM Tris-HCl, pH 8.0; 150 mM NaCl; 1% NP-40; 5 mM EDTA; 1X protease inhibitor cocktail, Pierce™, Thermo Fisher Inc., Shanghai, China). Cell lysates were sonicated and centrifuged at 4°C for 15 min. The supernatants were collected and measured for protein concentrations by using a BCA protein assay kit. After mixing with an equal volume of 2X loading buffer, samples were incubated at 95°C for 5 min. Equal amounts of cell lysates (15-20 μg/lane) were electrophoresed. After transfer to PVDF membranes, proteins of interest were sliced according to their masses and detected with their specific antibodies. The molecular markers above and below the band of interest were included. In case of two proteins of interest with close molecular masses, only one molecular marker was included in each blot. However, two blots were detected simutaneously. In some cases, the spliced slot was Antibodies for the detection of PB2 and NP proteins were diluted at 1:8000 in the antibody dilution buffer, antibodies against NS1 and β-actin were diluted 1:800, antibody against TRIF was diluted at 1:400, antibodies for other proteins were diluted at 1:2000. The blots were then probed with horseradish peroxidase-conjugated secondary antibodies, either anti-rabbit or anti-mouse IgG, followed by detection using the SuperSignal Western Pico enhanced chemiluminescence substrate (Pierce Chemical Co., Rockford, IL). The experiments were repeated at least three times with similar results. The specificity of the antibodies used to detect several key proteins was validated by gene knockout, overexpression, or the mutation of the phosphorylated amino acid. The density of the bands in Figure 8 was analyzed by using NIH Image-J software (NIH, Bethesda, MD, USA) (https://imagej.nih.gov/ij/) and normalized by the arbitrary units of β-actin or their total proteins as indicated. Quantified results were presented as the mean ± standard deviation (SD) derived, and these data were represented in bar graphs.

#### Recombinant DNA

Wild-type and kinase-dead (D138N) mRIPK1 were amplified from pBOB-mRIPK1 and pBOB-mRIPK1 D138N plasmids^55^ (kindly provided by Prof. Jiahuai Han, Xiamen University, China). The amplification products were then cloned into the Kpn I- and Nhe I-cleaved pCAGGS vector by using the Seamless Cloning and Assembly Kit (Transgen, Beijing, China). The pCAGGS-mRIPK1 and pCAGGS-mRIPK1 D138N plasmids were used to transfect RIPK1-knockout LET1 cells. The primer sequences utilized for PCR amplification please see Table S1.

#### Gene knockout

The gRNA sequences used to target the genes of interest were designed by using Benchling software. At least three sequences for each gene were selected. The sequences that successfully knocked out/down the expression of the genes in this study please see Table S2. The complementary oligonucleotides that contain 4 extra nucleotides at each end were annealed and ligated to the Bmsb I-digested LentiCRISPRv2 vector with the T4 DNA ligase. LET1 or NL20 cells seeded in a 24-well plate were transfected with the LentiCRISPRv2 vector or the vector encoding sgRNA by using the TurboFect transfection reagent following the manufacturer’s instruction. After incubation for 6 h, culture media were replaced with fresh complete media. Forty hours later, single cell suspensions were prepared, seeded in 6-well plates, and grown in the media containing puromycin (3 μg/ml for LET1 cells;1 μg/ml for NL20 cells). Fresh media containing the same concentrations of puromycin were changed every three days. After culturing for 3 weeks, individual clones were picked, expanded, and analyzed for TAK1, TRIF, RIPK1, ZBP1, RIPK3, caspase-8, caspase-3, GSDMD, GSDME, and TNFR expression by Western blot. At least two clones that lack the expression of TAK1, TRIF, RIPK1, ZBP1, RIPK3, caspase-3, caspase-8, GSDMD/E, and TNFR were used for investigating their role in IAV-induced cell death. Of note, knocking out GSDMD and GSDME were achieved by co-transfecting LET1 cells with the mixture of the LentiCRISPRv2 vector that encodes GSDMD or GSDME gRNA. Clones that were deficient of both GSDMD and GSDME were selected for the experiments in Figure 3.

#### Propidium iodide staining

LET1 cells seeded in 96-well plates were left uninfected or infected with H1N1 viruses (2 MOI) in the culture medium containing 2 μg/ml propidium iodide (Beyotime, Shanghai, China). The fluorescence intensity was measured at the indicated time using Infinite® 200 PRO microplate reader (Tecan, Switzerland). The percentage of cell death were calculated by dividing the arbitrary unit in each treatment with that in total cell death, which was obtained by treating the uninfected controls with 1% Triton X-100. The data represent the mean ± standard deviation (SD) of triplicate from one of three experiments with similar results.

#### LDH release assay

LET1 cells seeded in 96-well plates were left uninfected or infected with H1N1 viruses (2 MOI) in the culture medium for the indicated time. The plate was centrifuged, the conditioned media were collected and analyzed for LDH activity by using a Cytotoxicity Detection Kit (Sigma-Aldrich, St. Louis, MO, USA) following the manufacturer’s instruction. Data in bar graphs represents the mean ± SD of three independent experiments.

#### Fluorescent microscopy

LET1 cells seeded in 24-well plates were left uninfected or infected with H1N1 viruses (2 MOI) for the indicated time. The culture medium was removed and replaced with PBS containing 2 μg/ml propidium iodide. Images were captured using a Nikon or Olympus fluorescent microscope at 10x magnification. White light and fluorescence images were superimposed and processed by using the Adobe Photoshop software (Adobe Photoshop CC 2018).

### Quantification and statistical analysis

The band densities from 6 mice per group were analyzed using NIH Image-J software and normalized by the arbitrary units of their total protein bands or β-actin levels. The unpaired Student *t* test was used to determine the statistical difference between different groups. Repeated measure two-way ANOVA test was used to determine the statistical difference in cell death measured at multiple timepoints between wild-type and knockout cells. The *p* value of <0.05 was considered statistically significant. Normal distributions and statistics were performed with GraphPad Prism (GraphPad software 8.0.2) (https://www.graphpad.com/scientific-software/prism).
