# Supplementary material for: TRIF-TAK1 signaling suppresses caspase-8/3-mediated GSDMD/E activation and pyroptosis in influenza A virus-infected airway epithelial cells
Source: iScience. 2024 Dec 12;28(1):111581. doi: 10.1016/j.isci.2024.111581 (PMC11732511; doi:10.1016/j.isci.2024.111581)
Supplement: Document S1. Figures S1–S4 and Tables S1 and S2 [file mmc1.pdf]

iScience, Volume *28*

Supplemental information

TRIF-TAK1 signaling suppresses caspase-8/3-mediated GSDMD/E activation and pyroptosis in influenza A virus-infected airway epithelial cells

Yuling Sun, Huidi Yu, Zhihao Zhan, Wei Liu, Penggang Liu, Jing Sun, Pinghu Zhang, Xiaoquan Wang, Xiufan Liu, and Xiulong Xu

Supplementary Figures

Figure S1

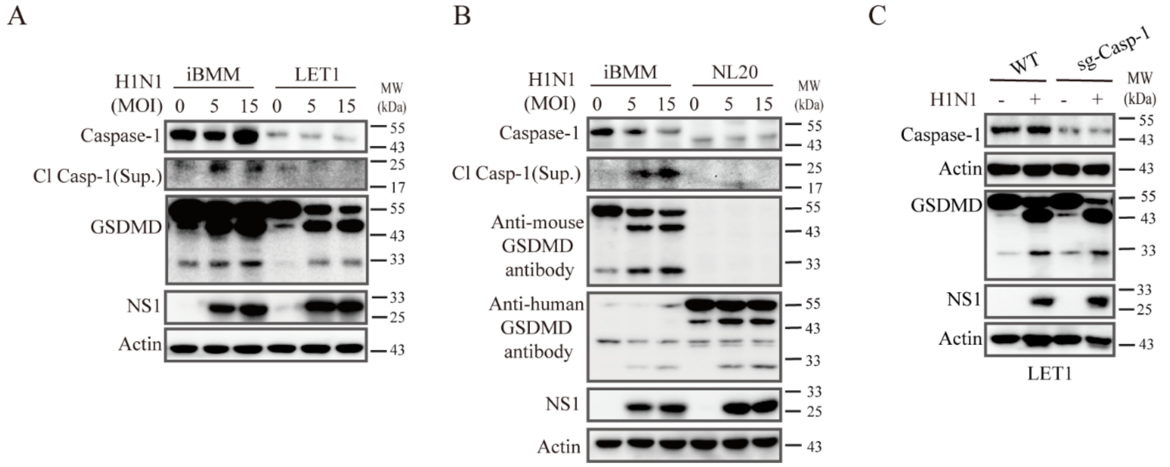

Figure S1. H1N1 induces cell death independent of Caspase-1, related to Figure 1. (**A**) iBMM and LET1 cells were infected with 5 and 15 MOI of H1N1 for 24 hr. Cell lysates were analyzed for the levels of caspase-1, GSDMD, NS1 and β-actin, whereas the conditioned media was analyzed for cleaved-caspase-1 by Western blot. (**B**) iBMM and NL20 cells were infected with 5 and 15 MOI of H1N1 for 24 hr. Cell lysates were analyzed for the levels of caspase-1, GSDMD, NS1 and β-actin, whereas the conditioned media was analyzed for cleaved-caspase-1 by Western blot. (**C**) Control and caspase-1 knockdown LET1 were infected with 2 MOI of H1N1 for 24 hr. Cell lysates were analyzed for the levels of caspase-1, GSDMD, NS1 and β-actin by Western blot.

Figure S2

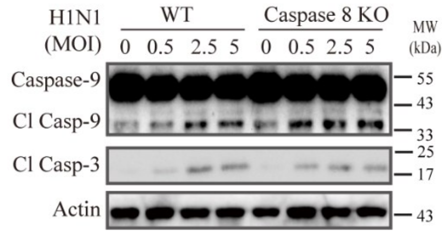

Figure. S2. Caspase-8 deficiency promotes caspase-9-mediated redundant activation of caspase-3 during H1N1 infection , related to Figure 2. Control and caspase-8 knockout LET1 were infected with 2 MOI of H1N1 for 24 h. Cell lysates were analyzed for the levels of caspase-9, caspase-3 and β-actin by Western blot.

Figure S3

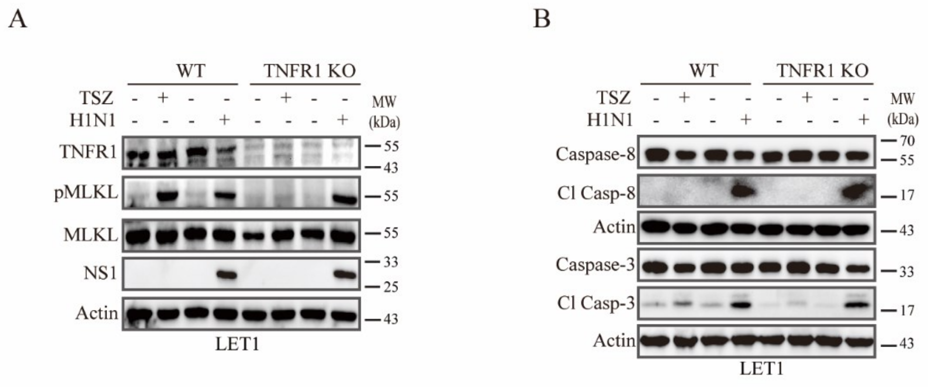

Figure. S3. H1N1 induces cell death independent of TNF-α, related to Figure 3. Control and TNFR1 knockout LET1 cells were infected with 2 MOI of H1N1 or stimulated with TSZ (30μM Z-VAD, 100nM Smac and 20ng/ml TNF-α) incubated for 14 hr. Cell lysates were analyzed for the levels of TNFR1, MLKL phosphorylation, caspase-8, cleaved caspase-8, caspase-3, cleaved caspase-3, NS1 and actin (**A & B**), by Western blot.

Figure S4

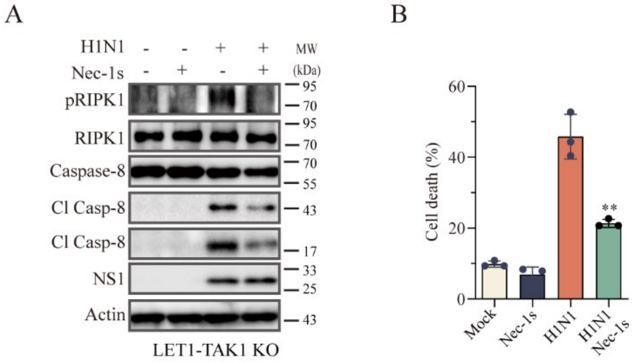

Figure. S4. Nec-1s inhibits H1N1-induced cell death in TAK1 deficient LET1 cells, related to Discussion. (**A & B**) TAK1 knockout LET1 cells were infected with H1N1 viruses (2 MOI) for 4 hr. Nec-1s (10 μM) was added and then incubated for another 8 hr. Cell lysates were analyzed for the levels of RIPK1 phosphorylation, caspase-8, cleaved caspase-8, NS1 and actin (**A**). Cell death was analyzed by reading in a plate reader at 12hr after infection (**B**).

Supplementary Tables

Table S1. Primers used for PCR, related to STAR Methods

Gene	Forward primer	Reverse primer
pCAGGS -mRIPK1	5'-CTCATCGATGCATGGTACCATGCAACCAGACATGTCCTTGGAC-3'	5'-GCAGAGGG-AAAAAGATCTGTTAGCTCTGGCTGGCACGAATCAAG-3'
pCAGGS-mRIPK1 D138N	5'-CTCATCGATGCATGGTACCATGCAACCAGACATGTCCTTGGAC-3'	5'-GCAGAGGG-AAAAAGATCTGTTAGCTCTGGCTGGCACGAATCAAG-3'

Table S2. gRNA target sequence, related to STAR Methods

Gene	Target sequence-1	Target sequence-2
mTAK1	5'-GATGATCGAAGCGCCGTCGC-3'	5'-TCGAAGTTCAGGACCTGCGA-3'
hTAK1	5'- GATGACTCGTTGTTGGTCTA-3'	5'-GAGTTGTTTGCAAAGCTAAG-3'
mTRIF	5'-TCTGGAACGCTAATTTCGTG-3'	5'-CAAGCTATGTAACACACCGC-3'
mRIPK1	5'-AGACAGCGGAGGCTTCGGGA-3'	5'-TGTGAAAGTCACGATCAACG-3'
mZBP1	5'-CAGGTGTTGAGCGATGACGG-3'	5'-GAAGATCTACCACTCACGTC-3'
mRIPK3	5'-GGAACCGCTGACGCACCAGT-3'	5'-TTCAGGGAGGGTCCCAGTCA-3'
mCaspase-3	5'-AGTGGACTCTGGGATCTATC-3'	5'-AATGTCATCTCGCTCTGGTA-3';
mCaspase-8	5'-GCAGGTCCCACCGACTGATG-3'	5'-CTTCCTAGACTGCAACCGAG-3'
mTNFR	5'-GCAGCAGGCCAGGCACGGTG-3'	5'-GATGGGGATACATCCATCAG-3'
mGSDMD	5'-CAGCATCCTGGCATTCCGAG-3'	5'-CAGAGGCGATCTCATTCCGG-3'
mGSDME	5'-GGGCTATTGGGACAGTCGTG-3'	5'-GTGTGAGAACCATAAGAGCG-3'
